# Polymorphism of CLE gene sequences in potato

**DOI:** 10.18699/VJ21.085

**Published:** 2021-11

**Authors:** M.S. Gancheva, M.R. Losev, A.A. Gurina, L.O. Poliushkevich, I.E. Dodueva, L.A. Lutova

**Affiliations:** Saint Petersburg State University, St. Petersburg, Russia; Saint Petersburg State University, St. Petersburg, Russia; Federal Research Center the N.I. Vavilov All-Russian Institute of Plant Genetic Resources (VIR), St. Petersburg, Russia; Saint Petersburg State University, St. Petersburg, Russia; Saint Petersburg State University, St. Petersburg, Russia; Saint Petersburg State University, St. Petersburg, Russia

**Keywords:** CLE genes, potato, Solanum bukasovii, Solanum verrucosum, Solanum commersonii, Solanum chaucha, Solanum curtilobum, Solanum juzepczukii, Solanum ajanhuiri, гены CLE, картофель, Solanum bukasovii, Solanum verrucosum, Solanum commersonii, Solanum chaucha, Solanum curtilobum, Solanum juzepczukii, Solanum ajanhuiri

## Abstract

CLE (CLV3/ESR) is one of the most important groups of peptide phytohormones: its members regulate the development of various plant organs and tissues, as well as interaction with some parasites and symbionts and response to environmental factors. In this regard, the identif ication and study of the CLE genes encoding the peptides of this group in cultivated plants are of great practical interest. Relatively little is known about the functions of CLE peptides in potato, since the CLE genes of the potato Solanum phureja Juz. et Buk. were characterized only in 2021. At the same time, potato includes plenty of tuberous species of the genus Solanum L., both wild and cultivated, and the diversity of its forms may depend on differences in the sequences of CLE genes. In this work, we performed a search for and analysis of the CLE gene sequences in three wild potato species (S. bukasovii Juz., S. verrucosum Schltdl., S. commersonii Dunal) and four cultivated species (S. chaucha Juz. et Buk., S. curtilobum Juz. et Buk., S. juzepczukii Juz. et Buk., S. ajanhuiri Juz. et Buk.). In total, we identif ied 332 CLE genes in the analyzed potato species: from 40 to 43 genes of this family for each potato species. All potato species taken for analysis had homologues of previously identif ied S. phureja CLE genes; at the same time, the CLE42 gene, which is absent from the S. phureja genome, is present in all other analyzed potato species. Polymorphism of CLE proteins of S. commersonii is signif icantly higher than that of other analyzed potato species, due to the fact that S. commersonii grows in places outside the growing areas of other potato species and this potato is probably not one of the ancestors of cultivated potato. We also found examples of polymorphism of domains of CLE proteins that carried different tions. Further study of potato CLE proteins will reveal their role in development, including regulation of productivity in this important agricultural crop.

## Abstract (ru)

CLE (CLV3/ESR) – одна из важнейших групп пептидных фитогормонов. Ее представители регулируют развитие различных органов и тканей растений, а также взаимодействие с некоторыми паразитами и симбионтами и ответ на факторы окружающей среды. В связи с этим идентификация и изучение генов CLE, кодирующих пептиды этой группы, у культурных растений представляют большой практический интерес. О функциях CLE пептидов у картофеля известно немного, поскольку гены CLE картофеля Solanum phureja Juz. et Buk. были охарактеризованы только в 2021 г. Вместе с тем картофель включает в себя много клубненосных видов рода Solanum L., как диких, так и культурных, и разнообразие его форм может зависеть в том числе от различий по последовательностям генов CLE. В этой работе мы впервые произвели поиск и анализ последовательностей генов CLE у трех диких видов картофеля (S. bukasovii Juz. et Rybin., S. verrucosum Schltdl., S. commersonii Dunal.) и четырех культурных (S. chaucha Juz. et Buk., S. curtilobum Juz. et Buk., S. juzepczukii Juz. et Buk., S. ajanhuiri Juz. et Buk.). У проанализированных видов картофеля выявлено 332 гена CLE: от 40 до 43 генов этого семейства для каждого вида картофеля. У всех видов картофеля, взятых в исследование, выявлены гомологи ранее идентифицированных генов CLE S. phureja; в то же время ген CLE42, отсутствующий в геноме S. phureja, найден у всех остальных проанализированных нами видов картофеля. Наибольшие отличия по аминокислотным последовательностям белков CLE оказались характерны для S. commersonii – вида, растущего вне ареалов культурных видов картофеля и, вероятно, не входящего в число их предков. Обнаружены также примеры полиморфизма по аминокислотным последовательностям доменов белков CLE, несущих разную функциональную нагрузку. Дальнейшее изучение белков CLE картофеля позволит выявить их роль в развитии этой важнейшей сельскохозяйственной культуры, в том числе в формировании признаков продуктивности.

## Introduction

The growth and development of higher plants, as well as their response to external stimuli, are regulated by intercellular communications mediated by phytohormones. In addition to the well-known and thoroughly studied “classical” plant hormones (IAA, cytokinins, ABA, etc.), numerous families of peptide hormones, which are mobile secreted oligopeptides or small proteins, play an important role in the coordination of plant development (Gancheva et al., 2019). One of the most famous families of peptide phytohormones with diverse functions is the family of CLE (CLV3/ESR) peptides. These peptides got their name from the first identified representatives: the CLAVATA3 (CLV3) Arabidopsis peptide (Clark et al., 1995) and the ENDOSPERM SURROUNDING REGION (ESR) maize peptide (Opsahl-Ferstad et al., 1997). Nowadays, genes encoding CLE peptides have been identified in all groups of terrestrial plants, as well as in the green alga Chlamydomonas reinhardtii (Oelker et al., 2008; Goad et al., 2017).

**Fig. 1.**
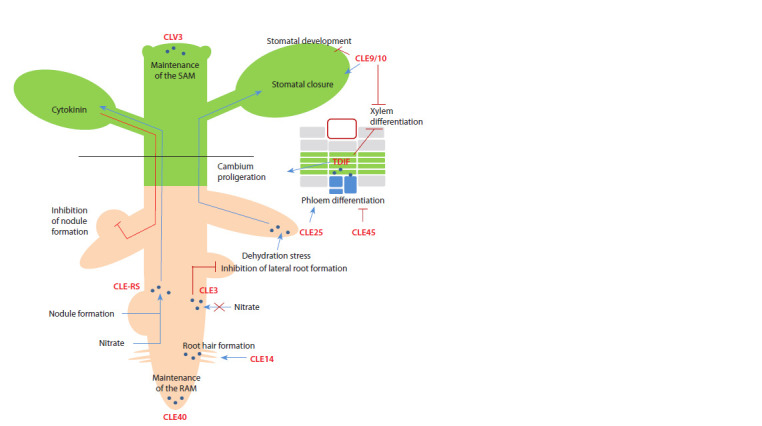
Some functions of CLE peptides in plant development SАМ – shoot apical meristem; RАМ – root apical meristem.

CLE genes encode proteins 100–150 amino acids (AA) long, which have a signaling domain (SD) at the N-terminus, a conserved CLE domain at the C-terminus, and a variable domain (VD) between them (Strabala et al., 2014). The CLE domain, consisting of 12 AAs, is a functional part of the CLE protein: immediately after synthesis, the precursor protein undergoes proteolytic processing and post-translational modifications (Kondo et al., 2006; Ni et al., 2011). As a result, what remains of it is the CLE domain with modifications (hydroxylation, arabinosylation) of conservative proline residues; this is the mature CLE peptide. CLE peptides that are secreted into the intercellular space become ligands for receptor kinases of Leucine Reach Repeats containing Receptor- Like Kinases (LRR-RLK) families and CRINKLY4 that are located on the plasma membranes of cells (Poliushkevich et al., 2020). By interacting with receptors, CLE peptides trigger a signaling cascade, the targets of which are homeodomaincontaining transcription factors of the WOX family that regulate the maintenance of stem cell niches in plants (Tvorogova et al., 2021). The known functions of CLE peptides include control of shoot and root apical meristems and cambium activity, differentiation of vascular tissues, formation of lateral roots and nodules, early embryogenesis, stomatal development, and response to several environmental factors: water availability and changes in soil nitrogen composition (Yamaguchi et al., 2016; Fletcher, 2020) (Fig. 1)

In all angiosperm species studied, the CLE peptides are encoded by numerous genes. For instance, in the relatively small genome of Arabidopsis thaliana, there are 32 CLE genes (Sharma et al., 2003; Strabala et al., 2006), and each A. thaliana CLE gene is characterized by a unique spatial pattern of expression (Jun et al., 2010). However, some CLE genes encode the same CLE peptides. It is suggested that such an excess of CLE peptides is necessary for fine regulation of plant development (Kinoshita et al., 2007).

It is obvious that the family of CLE genes is not limited by the genes discovered to date. The accumulation of genomic data and computer software improvement make it possible to identify new members of this family. In our study, in the reference potato genome, which is the sequence of the doubled monoploid clone Solanum phureja DM-1-3 516R44 (Gancheva et al., 2021), 41 CLE genes that encode 37 unique CLE peptides were identified. Besides, many cultivated and wild-growing potato species are known. According to various authors, potatoes (Solanum L., section Petota Dumort.) include 112 to 235 species (Huamán, Ross, 1985; Spooner et al., 2014). The genomes of some potato species are currently sequenced and available in genomic databases.

In our work, we searched for and analyzed CLE genes in the genomes of seven potato species: three wild species (S. bukasovii, S. verrucosum, S. commersonii) and four primitive cultivated species (S. chaucha, S. curtilobum, S. juzepczukii, S. ajanhuiri). All genomes are provided in the NCBI database. In total, we found 332 CLE genes and identified unique peptides in individual potato species that can perform other unknown functions or be completely nonfunctional. In addition, we found similarities in the sequences of different CLEs, which may indicate their common genetic origin.

## Materials and methods

We used the genome assemblies of various potato species presented in the NCBI database: wild species S. commersonii, S. verrucosum, and S. bukasovii, along with primitive cultivated species S. chaucha, S. juzepczukii, S. curtilobum, and S. ajanhuiri. In the present work the system of J. Hawkes was used.

Solanum commersonii is a widespread diploid potato species in South America, present at the coastal zone of the Atlantic Ocean, mainly located in Argentina and Uruguay. Its natural habitat extends from sea level to an altitude of 1300 m. S. commersonii is ruderal, and its primary habitats are rocky areas, dunes, and growing areas of cultivated plants (Hawkes, Hjerting, 1969).

Solanum verrucosum is a diploid potato, which, unlike other species studied in this work, is widespread in North America, more precisely in Mexico. Yet, it is believed that S. verrucosum is evolutionarily closer to the ancestors of cultivated potato species than S. commersonii (Hawkes, 1990). The habitats of this species are woodlands

Solanum bukasovii is a diploid South American potato species that grows at an altitude of 3300–4000 m above sea level in Peru. It belongs to the group of wild potato species from which cultivated potato species are believed to have evolved (Li et al., 2018).

Solanum juzepczukii and S. curtilobum are alpine triploid and pentaploid potato species, respectively, that grow in a very limited area. They belong to the group of “bitter” potatoes due to the high content of glycoalkaloids. Only some of their clones were found to be cultivated in the highlands of Peru and Bolivia, where other types of potatoes cannot be grown due to the conditions. They are more resistant to frost than any other domestic potato species (Lekhnovich, 1971).

Solanum ajanhuiri is a diploid alpine species. It is cultivated at an altitude of more than 3900 m in the area of Lake Titicaca. S. ajanhuiri is also hardy, but unlike S. juzepczukii and S. curtilobum, it has a significantly lower glycoalkaloids content in tubers (Hawkes, 1990).

Solanum chaucha is a triploid species found mainly in the northern mountainous regions of South America (Ecuador, Colombia) and northern Peru. It is cultivated at lower altitudes than the above-mentioned cultivated potato species (Hawkes, 1990).

The search for CLE genes in different potato species was carried out according to homology with genes from the CLE family in S. phureja (Gancheva et al., 2021), A. thaliana (Sharma et al., 2003; Strabala et al., 2006), and tomato Solanum lycopersicum (Zhang et al., 2014; Gancheva et al., 2021) using the Nucleotide Basic Local Alignment Search Tool (BLASTN) and the discontiguous megablast algorithm (Altschul et al., 1990) in the NCBI database (https://blast.ncbi. nlm.nih.gov/Blast.cgi), where genome assemblies of all the studied potato species are available (see the Table). The alignment of amino acid and nucleotide sequences was carried out using the Muscle algorithm in the MEGA7 program (https:// www.megasoftware.net/) (Kumar et al., 2016).

Phylogenetic analysis was performed in the MEGA7 program using the “nearest neighbour” method (Saitou, Nei, 1987) with default settings and a bootstrap of 1000 (Felsenstein, 1985). CLE protein signaling domains were predicted in the SignalP-5.0 program (http://www.cbs.dtu.dk/services/ SignalP/). Consensus sequences of CLE proteins were visualized in the Geneious Prime software (https://www.geneious. com/features/).

## Results

Using the NCBI database, we identified CLE genes in seven potato species by homology with the CLE genes of potato S. phureja (SphCLE), arabidopsis (A. thaliana), and tomato (S. lycopersicum). Species being studied were primitive domestic species S. chaucha, S. curtilobum, S. juzepczukii, S. ajanhuiri, and wild species S. bukasovii, S. commersonii, S. verrucosum (Supplementary Material)1. We also identified CLE genes in the primitive cultivated potato S. stenotomum and in the wild species S. pinnatisectum. However, due to the insufficient quality of the genome assemblies these genes were not selected for further analysis

For each potato species, a different number of CLE genes was found, 40 to 43 (see the Table). This is primarily due to the different qualities of genome assemblies. For instance, the genome of S. verrucosum is published in the form of extended scaffolds, while the assembly of the S. curtilobum genome is presented at the contig level. As a result, some S. curtilobum CLEs were not included in the analysis due to contig breaks and the inability to analyze the entire gene sequence. Homologues of most previously identified CLE genes in S. phureja (SphCLE) (Gancheva et al., 2021) were found in all potato species analyzed. Furthermore, the gene that we named CLE42 was not found in the S. phureja genome, although it is present in all other potato species studied.

**Table:**
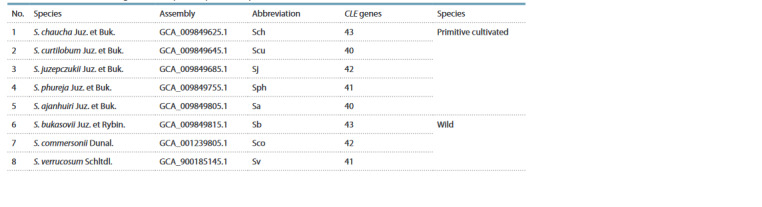
The number of identif ied CLE genes in the potato species analyzed

**Fig. 2.**
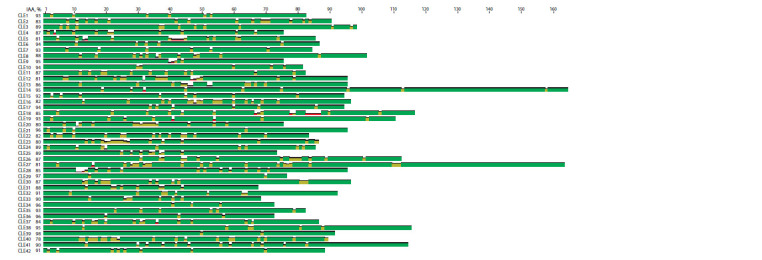
Consensus sequences of CLE proteins from 8 potato species. IAA – percentage of identical AA.

**Fig. 3.**
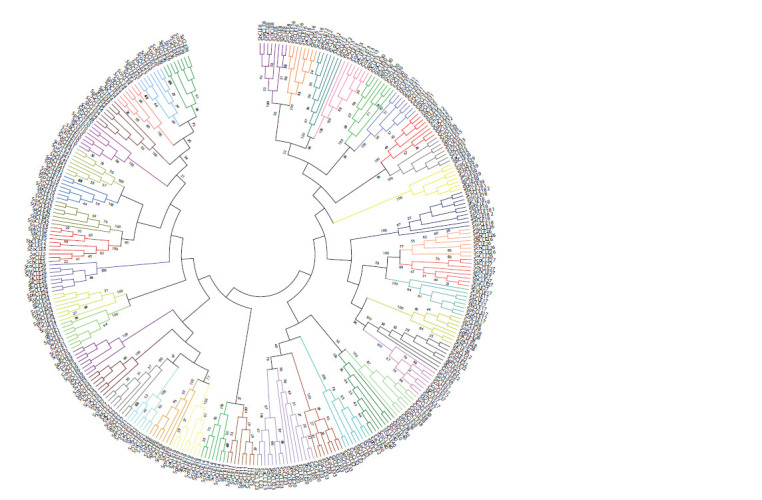
Phylogenetic tree of potato CLE proteins.

Analyzing the amino acid sequences (AAS) of individual CLE proteins in different potato species, we found a high percentage of their similarity (78–98 % identical AA) (Fig. 2). Moreover, there are both completely identical AAS of proteins (for example, the CLE39 AASs are identical in six of the eight analyzed species) and variants with no identical AASs among the species (CLE3, CLE16, CLE40). However, the CLE proteins in the studied potato species are very similar to each other. Based on the complete protein sequence, individual CLEs form groups, each of which includes all homologues of one CLE protein from different potato species (Fig. 3). Among all analyzed potato species, the CLE proteins of S. commersonii contain a large number of unique AAs, which are absent in other species.

CLE proteins include three domains: the signaling domain (SD), the variable domain (VD), and the CLE domain. The CLE domain is the most conserved functional part of the protein, and substitutions are rare here. In other protein domains, substitutions occur much more frequently. Although substitutions in the CLE domain will bring the greatest effect on the phenotype, SD and VD are also important and are involved in the implementation of CLE peptide function. SD and VD are active in the processing of CLE peptides and can thus influence the availability of mature peptides in certain cells and tissues (Meng et al., 2010). It turned out that, in different potato species, AASs of CLE domains are almost identical within each group. At the other extreme, the nucleotide sequences (NSs) of CLE domain in proteins of the same group vary between species. For example, proteins of the CLE12 group, although they have identical AASs of the CLE domains, differ in the NSs of the corresponding gene regions.

**Fig. 4.**
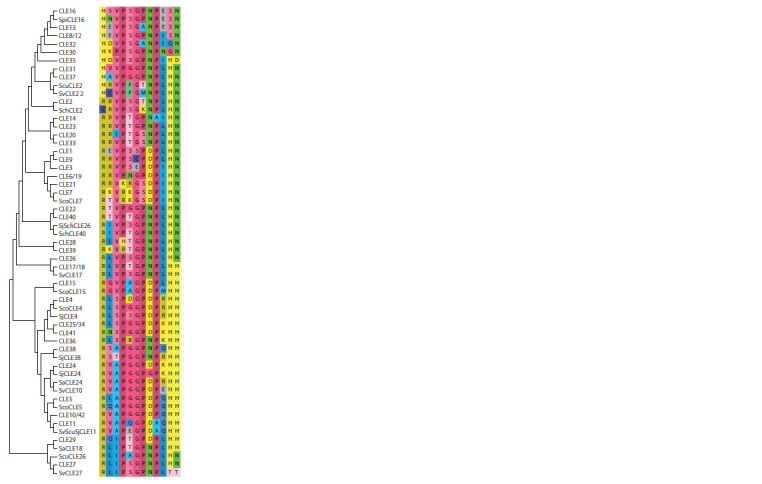
Phylogenetic tree and alignment of unique potato CLE peptides.

Although we identified 41 CLE genes in S. phureja, they encode 37 CLE peptides (Gancheva et al., 2021). This is because the AASs of CLE domains are identical in some proteins (for example, in CLE8 and CLE12). However, in other potato species, 20 additional CLE domains were found,which are absent in S. phureja. All these domains are similar to the CLE domains of S. phureja proteins, but differ from them by 1–4 AA (Fig. 4). 

As mentioned above, in some CLE proteins of the same plant species, CLE domains are identical (for example, CLE41 and CLE44 domains of the arabidopsis). In potato, 10 pairs of such proteins with identical CLE domains were found. Mature peptides that have identical AASs of CLE domains are peptides from CLE10 and CLE38 groups, CLE17 and CLE18, CLE25 and CLE34, CLE6 and CLE19, CLE8 and CLE12. However, the remaining AASs of these proteins (outside the CLE domain) vary significantly within each pair.

At the same time, some potato CLE proteins have a similar AAS, but their CLE domains are not identical and differ by one or several AAs (for example, CLE32 and CLE35, CLE26 and CLE27). Interestingly, some proteins are grouped differently depending on whether the CLE domain or the rest of AAS of the protein is being compared. For instance, CLE37 is closer to CLE31 (they differ by 1 AA) when comparing their CLE domains. However, the rest of the CLE37 protein forms a group with CLE22 protein. The same thing happens in the case of CLE25 and CLE34 proteins. These proteins have identical CLE domains, but their sequences outside the CLE domain are different and, comparing them, CLE34 is grouped with CLE36, not CLE25. Sometimes such a scenario occurs only within one potato species. For example, the CLE domain of ScoCLE4 differs by only 1 AA from CLE25 (while in other potato species CLE domains of CLE4 differ by 2 AA from CLE25); notably, protein sequence outside the CLE domain belongs to CLE4. Obviously, differences in the sequence of the CLE domain (and, consequently, of the mature peptide) should lead to the functional diversity of CLE proteins, despite the great similarity of their AASs. CLE proteins, similar in AASs, but distinct in the CLE domain, are also found in other plant species (for example, CLE41 and CLE42 or CLE25 and CLE26 of the arabidopsis). Their genes may have emerged as a result of duplications with subsequent mutations in the sequence of the CLE domain, which led to the emergence of new functions (Yaginuma et al., 2011; Takahashi et al., 2018). Indeed, some CLE genes in potatoes are duplicated, and their sequences weakly vary from each other. For instance, in all analyzed potato species, CLE26 and CLE2 genes are duplicated. Sometimes such duplicated genes have substitutions in CLE domain sequence, which leads to the emergence of a unique peptide (for example, SvCLE2-2). However, due to the high level of similarity, such genes are not counted in the Table.

Sometimes, CLE domains of some CLE proteins are identical in several potato species and differ from CLE domains of the same proteins in other species. For example, in the CLE26 proteins of S. juzepczukii and S. chaucha, I is in the second position of the CLE domain, while in other species there is L. The CLE11 proteins of S. verrucosum, S. curtilobum, and S. juzepczukii, in the fifth position of CLE domain have E, while in other species there is Q (see Fig. 4). The largest number of unique CLE domains, which differ from all other potato species studied, was found in S. verrucosum, S. commersonii, and S. juzepczukii.

**Fig. 5.**
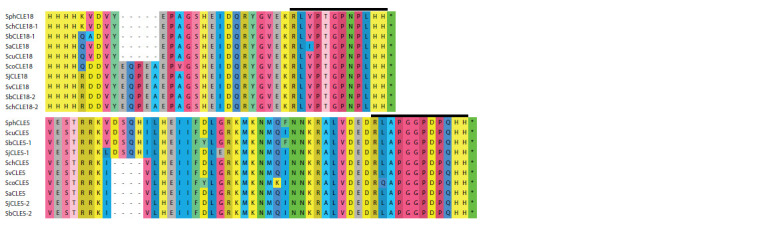
Fragments of CLE18 and CLE5 protein alignments. CLE domain is highlighted with a black line.

Great interspecies differences in the AASs of CLE proteins relate to sequences outside the CLE domain. Thus, among the genes of the CLE8 group, SvCLE8 stands out, which has three additional nucleotides in the VD; it makes the protein 1 AA longer. CLE18 proteins of S. verrucosum, S. commersonii, S. bukasovii, S. chaucha, and S. juzepczukii have a region 5 AA in the VD, while CLE18 of other species does not have such a region. At the same time, S. bukasovii and S. chaucha each have two CLE18 genes encoding proteins with and without this region (Fig. 5). Asimilar situation occurs for the CLE5 protein: a region 4AA in theVD is present in S. phureja, S. curtilobum, S. juzepczukii, S. bukasovii, but absent in S. chaucha, S. verrucosum, S. ajanhuiri, S. commersonii, whereas S. bukasovii and S. juzepczukii each have two CLE5 genes encoding proteins without this region and with it (see Fig. 5). Sometimes, great differences in the AASs of CLE proteins are observed only within one species. For example, protein SvCLE30, in contrast to CLE30 of other potato species, lacks 3 AAs in the VD. In the VD of ScoCLE20, there is no fragment of 8 AAs, and its SD is lengthened by 1 AA. S. juzepczukii has two CLE12 genes, and one of them encodes a protein that differs from CLE12 of other species in that the onset of its SD is extended by 6 AAs, while 3 AAs are absent in the VD.

In sum, the analysis of CLEs nucleotide and amino acid sequences of the potato species studied revealed several examples of polymorphism in different regions of CLE carrying different functional loads. This polymorphism can affect the activity of CLE peptides. The differences relate to CLE domain itself, from which the actual CLE peptide is formed, or to sequences outside it, which can affect the processing of CLE protein. At the same time, accurate data on the functional difference between CLE proteins in potato species can be obtained only with a precise analysis of their functions. For example, in overexpression experiments of the corresponding genes or plant treatment with synthetic CLE peptides of different potato species. Our research can serve as the groundwork for further research in this area.

## Discussion

In this research, we performed a search and analysis of the genes encoding CLE proteins in different species of potatoes: wild and primitive cultivated. Of the 332 identified CLE genes, we found 57 genes that encode unique CLE peptides. In total, we identified 42 genes that are present in almost all potato species analyzed. At the same time, there are genes that are very similar to each other in different species and those that have significant interspecific differences. Thus, in CLE genes of S. commersonii, presumably, the largest number of unique substitutions occurred, which led to serious differences in its CLE peptides compared with those in other potato species. S. commersonii is a wild potato that differs from the rest of the species analyzed by its habitat. It grows outside the growing areas of cultivated potatoes and, most likely, is not among the ancestors of cultivated potatoes (Juzepczuk, Bukasov, 1929).

Some of the revealed differences in the sequences of CLE proteins are unique for a certain species of potato, while other differences relate to several ones. Of special interest are the proteins CLE5 and CLE18, which vary in the presence or absence of a region of 4–5 AA. At the same time, there are potato species in which both protein variants are present (see Fig. 4), which may be associated with the natural hybridization of potatoes (Hawkes, 1990).

Additionally, in some potato species, substitutions occurred in CLE domain, which could affect the functions of the corresponding peptides. For example, divergence in 1 AA in CLE domain of A. thaliana CLE peptides results in the divergence of their functions: one peptide is involved in the response to water shortage (CLE25), while the other is not (CLE26) (Takahashi et al., 2018). Unique peptides that appear in individual potato species due to the differences in CLE domain can perform distinct functions or completely lose functionality. Furthermore, changes in the sequence outside the CLE domain can also affect the functioning of the CLE peptide and, in different potato species, changes in VD or SD may affect peptide activity. At the same time, the sequence similarity of different CLEs may point to their common origin. The presence of duplicated genes, such as CLE2-2, in which substitutions occur in CLE domain sequence and which may subsequently lead to the emergence of new genes confirms this hypothesis.

## Conclusion

In summary, we found that CLE proteins in various potato species are similar; however, they also have differences that could affect their functioning. Further study of CLE proteins will reveal their role in potato development.

## Conflict of interest

The authors declare no conflict of interest.
